# Monthly Memoranda

**Published:** 1854-01

**Authors:** 


### EDITORIAL DEPARTMENT.

### MEDICAL MEMORANDA.

*Inunction in Scarlatina.*—The attention of the profession has been called to this subject, and much speculation has been indulged in relative to its therapeutic powers. Some have regarded it inoperative, empirical, and nothing more than an old wives' fable. Others have claimed that it possesses powers most potent in the control of that disease, and claim also that their opportunities of observation have been sufficiently ample to form an opinion as to its virtues. While this controversy has been going forward, we were by no means disinterested spectators, as we have witnessed several epidemic visitations, more disastrous in their results, in the localities in which they prevailed, than the inhuman edict of Herod itself. We have examined the testimony *pro and con,* and are constrained to say that it preponderates in favor of its utility, not only on account of the number, but the respectability, of the witnesses. Not disposed to pin our faith or belief to the *ipse dixit* of either party, we have been on the road of inquiry ourselves, but the number of cases in which we have made the trial, is too limited on which to predicate an opinion. So far, however, as they go, they are decidedly in favor of its use, and sufficiently encouraging to induce us to resort to it again when a case presents itself.

Those who oppose it because of its inutility, have given no "good and sufficient reason" for their opinion, but have stood off, at a respectful distance, and hurled their missives at it. Its advocates, on the other hand, have given us no clue to its *modus operandi,* being satisfied with the practical effects or results. And thus it stands. We would suggest a few questions barely with the view of setting in motion the thoughts of all parties.

We would then enquire, first, whether there is not in scarlatina an arrest of the secretion, on the part of both the sudoriparous glands and the sebaceous follicles? Does not the secretion from the sebaceous follicles contain animal matter, oil, and stearine? Does not the application of lard supply to the skin, the product of these glands, and exercise its physiological office, in the absence of the normal secretion, in relaxing the skin and in favoring the transpiration from the sudoriparous glands?

In all our experience in febrile diseases, we have never witnessed a higher temperature of the skin, nor one more especially deserving the use of the term *parched* to convey a correct idea of its condition than exists in scarlatina. The use of inunction, in the few cases in which we have tried it, always diminished the heat of the skin, and in one or two cases perspiration followed.

If then, the sabraceous glands secrete oil and stearine for the protection, as it is supposed, of the skin from the injurious influences of moisture, why is it that, usually and generally, scarlatina prevails in cold damp localities and in the spring seasons of the year? If there be a total suspension of the transpiration from the skin, we can readily account for the exaggerated character of the symptoms, for who could endure for any length of time, that every pore be closed by an impenetrable and impermeable coating over the surface of the body?

There are some phenomena connected with scarlatina maligna which have never been explained to our satisfaction. In its pathological manifestations it certainly is analagous to epidemic erysipelas, or what is termed "black tongue."

*Vesico-Vaginal Fistula.*—The peculiar character of this most unfortunate difficulty, has called forth the best efforts of the best surgeons to cure and relieve, but the results have neither been fortunate to humanity nor flattering to the department of surgery. When any method, which gives promise of certain and permanent relief is proposed, it is eagerly seized upon under the hope that a means of relief has at last been discovered. These hopes, owakened from time to time, have in many instances proved delusive. A new idea, however, has been awakened through the skill and perseverance of Dr. Sims, of N.Y., formerly of Alabama. His pamphlet, in which he gives a lucid description of his operation, the instruments and appliances in its performance, and the various steps in its progress, is now before us. By the aid of diagrams, in addition to the description, it is easy to comprehend the mode of procedure and appreciate the superiority of the plan over other modes of operating. We thank the author for the promise which his method offers of an escape from this loathsome and distressing affliction. If in the future his plan shall prove as successful as it has already been in his hands, he will deserve to be ranked among the benefactors of his kind.

The suture he adopts to secure the coaptatio of the edges of the fistulous opening, (already pared with the knife,) he calls the *clamp suture*, which consists of two polished bars of lead or silver, perforated with one, two, three, and sometimes four, holes, for the passage of the silver wire. The bars are but one line in diameter and the wire but the size of a horse hair, which is toughened by annealing it. The wire is preceded by a silken thread, to which it is attached and by which it is drawn through the punctures made by the needle in the edges of the fistulous orifice. The distal ends of the wire are drawn through the perforations in one of the jaws of the clamps and the wire passed around the clamp and fastened, or a perforated shot is passed upon the wire and drawn close up to the jaw of the clamp. The proximal ends of the wire are then drawn until the clamp is brought against the side of the fistulous opening farthest distant from the operator. This done, the proximal clamp is placed upon the proximal ends of the wire, and the perforated shot put on each wire, behind the clamp; both are then carried up to the edge of the opening nearest the operator and there confined.

This constitutes the chief feature of the operation, to perform which the position of the patient, the kind of speculum, and the mode and the manner of using it, are essential requisites. But the entire process cannot be intelligently conveyed here without the benefit of diagrams. Dr. Sims began his enquiries in 1849, since which he has been faithfully and diligently prosecuting them, when his health, which was delicate, permitted. He has escaped from the deteriorating climate of the South and has taken up his residence in New York, where he will devote his time to the prosecution of this branch of surgery.

Prof. Mussey, of Cincinnati, has recently and successfully operated for recto-vaginal fistula, and used the *clamp suture* of Dr. Sims, of which he speaks in terms of high commendation and praise. A description of his plan of operating appeared some time since in one of the medical journals of Philadelphia; but so far as we are aware no one, except in the instance of Prof. Mussey, has adopted the suture.

*Ether in Capsules.*—A paragraph is going around among the Medical Journals regarding the administration of ether in capsules. A French writer states that he has for some time been accustomed to give ether in capsules in nervous affections. After several years of observation, by himself and others, he concludes that ether, when introduced in a known dose, pure, and without loss, into the stomach, has an effect which was totally unknown until the preparation of the ether-pearls (*perles d'ether.*) According to the old plan, the ether became partly volatilized before passing half way down the œsophagus; and what arrived in the stomach was dissolved in water, and in a state favorable to rapid and sudden volatilization. M. Clertan has several times seen neuralgia, hemicrania, and gastralgia, arrested instantaneously by from one to three of these capsules; while ether draughts, and ether in syrup, had been largely given without any effect.

The editor of the *Union Medicale* for April 12th, in noticing this preparation, states that the ether capsules are already employed extensively by M. Trousseau, M. Pidoux, and other practitioners in Paris. The advantages of the capsules are:

1. The ether can be administered in a known dose—each capsule containing four or five drops.

2. The capsules are inodorous; so that ether can, without their knowledge, be given to persons to whom its smell is repulsive.

3. The capsules permit neither evaporation nor decomposition of the ether; they may be kept a year at least, or indefinitely, according to M. Clertan.

4. The ether arrives in the stomach without irritating the membrane of the mouth or pharynx, or producing cough; and it produces its sedative action by its rapid absorption.

*Irritable Stomach.*—The *Nashville Journal of Med. and Surg.* says that Dr. Robertson, of Tennessee, stated that for a great number of years, he has compelled the stomach to retain laudanum in cholera morbus by grasping the little finger of the patient, and forcibly and *painfully* flexing the third phalanx upon the second. The medicine was swallowed while the grasp was firmly retained, which was continued for a few moments afterwards. "They have tried to knock me down," said the venerable physician, "but I defied them to throw up the medicine."

*Creosote.*—The Virginia Medical Journal quotes from a foreign Review as follows: "M. Arendt states that the great advantage he had derived from the use of creosote in asthma and bronchitis, had induced him to try it in other affections, especially those of the mucous membranes. In chronic varicose ophthalmia he had found from 1 to 3 drops of creosote to an ounce of water a valuable collyrium. Cardialgia, especially the idiopathic form in women, was speedily amenable to creosote, 3 drops in sugared water relieving the severest pain, a repetition in two or three hours being rarely required. Leucorrhœa often yielded to a lotion of 2 drops to an ounce, thrown in two or three times a day. So also three or four injections usually sufficed for the cure of gleet. In menorrhagia in non-pregnant women, and in some cases of hæmorrhage before delivery, depending on placenta prævia, it has been found very useful. Indeed, it is a valuable hæmostatic agent, whenever the bleeding proceeds from small vessels, and especially those of the mucous membranes. In some of these cases a more concentrated mixture is required, as 10 to 20 drops to the ounce.

*Felt Splint.*—Dr. Hamilton gives, in the Buffalo Med. Jour., a description of the method of making what he terms the best splint ever used. We recommend it to our surgical readers: "Dissolve three pounds of gum shellac in two quarts of alcohol. It should be dissolved in a tin vessel, furnished with a tight cover to prevent evaporation. Spread a piece of old or new woolen cloth on a board, and with a clean brush saturate both sides of the cloth with the solution. Hang it up until it is thoroughly dried. Lay it again upon the board and apply a second coat of the solution to one side only of the cloth. Dry again, and apply a third coat to the same side. There will now be three successive layers upon one side and one on the opposite. While the last coat is yet fresh, fold the cloth so that the side having three coats shall be applied to itself. Now with a hot flat-iron, smooth and press the surfaces together. When it is cold, a slight rubbing with sand-paper makes it fit for use. It becomes a firm, almost unyielding board, but exposure to a moderate heat will make it pliant, so that it can easily and accurately be adapted to any surface."

